# The Virginia Springs, with Their Analysis, and Some Remarks on Their Character; Together with a Directory for the Use of the White Sulphur Water, and Account of the Diseases to Which It Is Applicable

**Published:** 1847-07

**Authors:** 


ART. VII.—*The Virginia Springs,* with their Analysis, and some Remarks on their Character; together with a Directory for the Use of the White Sulphur Water, and an Account of the Diseases to which it is applicable. To which is added a Review of a Portion of Wm. Burke's Book on the Mineral Springs of Western Virginia, etc., and an Account of the different Routes to the Springs. By JOHN J. MOORMAN, M.D., Resident Physician at the White Sulphur Springs. 12mo, pp. 219. Philadelphia: Lindsay and Blakiston: 1847.

This little volume begins with a notice of the use of mineral waters by the Greeks and Romans, who seem to have placed a high estimate on their remedial virtues. Hippocrates was acquainted with various mineral Springs, and Pliny, in his Natural History, mentions a number the efficacy of which had been tested in his day.

The first satisfactory analysis of mineral waters was executed by a commission of the French Academy of Sciences in 1670. Since that time analytical processes have been greatly improved, and the chemist now is able to determine the relative proportion and absolute quantity of every ingredient with the utmost precision. It is evident that the medicinal action of mineral springs must vary with their constitution, and that the indiscriminate use of their waters must be attended with injury. The object of Dr. Moorman in this volume is to direct the public to a judicious use of these agents, in the study of which he has spent a number of years.

The *White Sulphur Springs* are among the most noted in the United States, and a very general interest must be felt in a knowledge of their composition and qualities. According to the most recent analysis of this water by Prof. W. B. Rogers, 100 cubic inches, or 3½ pints, contain 65.54 grains of solid matter. The quantity of each of the ingredients is shown in the following table:

| | | | |
|---|---|---|---|
| Sulphate of lime | 31.680 grs. | Chloride of sodium | 0.226 grs. |
| Sulphate of magnesia | 8.241 " | Proto-sulphate of iron | 0.069 " |
| Sulphate of Soda | 4.050 " | Sulphate of alumina | 0.012 " |
| Carbonate of lime | 1.530 " | Earthy phosphates | a trace. |
| Carbonate of magnesia | 0.506 " | Azotized organic matter blended with a large proportion of sulphur, about | 5 " |
| Chloride of magnesium | 0.071 " | Iodine, combined with sodium or magnesium. | |
| Chloride of calcium | 0.010 " | | |

The gaseous contents are as follows: Sulphuretted hydrogen, 0.66 to 1.30 cubic inches; nitrogen, 1.88; oxygen, 0.19; carbonic acid, 3.67.

The directions laid down by Dr. M. for the use of these waters may be safely followed by the valetudinary, founded as they are upon much experience and close observation. His book ought to be in the hands of every invalid at the springs.

Dr. M. relates the following curious fact relative to the secondary formation of sulphuretted hydrogen:

"In a shipment of this water to Calcutta, some years since, the 'transporting company' had the water bottled in Boston, from barrels that had been filled at the spring six months before. This water, although tasteless and inodorous when put into the bottles at Boston, was found, on its arrival at Calcutta, so strongly impregnated with the hydro-sulphuric acid gas as to render it necessary, under the direction of an intelligent gentleman of Boston, who had witnessed this secondary formation of gas before, to uncork the bottles for some time before using, that the excess of gas might escape."

He says he has observed the same thing when sulphur water has been thawed in a warm room, and he conjectures that some part of the efficacy of sulphur water may depend upon changes which take place in it after it has reached the stomach. Nothing is more true than his remark, that "experience is the only sure guide in the administration of mineral waters."

The *Blue Sulphur Spring* contains the following ingredients: Sulphate of lime, sulphate of magnesia, sulphate of soda, carbonate of lime and of magnesia, chloride of calcium, of magnesium and sodium, hydrosulph. of sodium and magnesium, oxide of iron existing as protosulphate, iodine, sulphur, organic matters, sulphuretted hydrogen, carbonic acid, oxygen and nitrogen.

The *Salt Sulphur Springs* contain sulphate of lime, of magnesia and soda, carbonate of lime and of magnesia, chloride of magnesium, of sodium and calcium, iron, organic matter, earthy phosphates and iodine, besides the gases above enumerated.

The *Red Sulphur Springs* contain sulphuretted hydrogen, carbonic acid, nitrogen, sulphate of lime, of soda and magnesia, carbonate of lime and muriate of soda.

The *Sweet Springs* contain sulphate of magnesia and of lime, chloride of sodium and of calcium, iron and carbonic acid.

The *Sweet Chalybeate Spring* contains carbonate of lime, of magnesia and iron, silex, sulphate of magnesia, of soda and lime, muriate of soda, iodine, carbonic acid, nitrogen, hydrogen and sulphuretted hydrogen.

The two last named springs are in repute for their tonic properties, but as chalybeate springs are common in all parts of the country, there is no reason why invalids should travel all the way to Virginia in pursuit of their invigorating virtues. Iron is readily detected in water by nutgalls, and any one may satisfy himself of its presence by this test. Where it exists in very minute quantity, instead of using an infusion of galls, it is better to suspend a fragment of gall by a thread in the water; a purple tinge is given to the water if iron be present. Chalybeate springs possess great efficacy in diseases marked by an impoverished condition of the blood, that form being often the best in which iron can be administered. We have now vividly in our memory a case of anasarca cured in Tennessee, a good many years ago, by a chalybeate spring. The subject was a woman, about forty years of age, in whom the disease had been induced by profuse uterine hemorrhage. The spring was within two miles of her residence, and she rode to it on horseback every morning. Hers was a case in which the recovery must be attributed to the efficacy of the water alone.

